# Investigation of the *N*-Terminus Amino Function of Arg_10_-Teixobactin

**DOI:** 10.3390/molecules22101632

**Published:** 2017-09-28

**Authors:** Shimaa A. H. Abdel Monaim, Sikabwe Noki, Estelle J. Ramchuran, Ayman El-Faham, Fernando Albericio, Beatriz G. de la Torre

**Affiliations:** 1 Catalysis and Peptide Research Unit, School of Health Sciences, University of KwaZulu-Natal, Durban 4001, South Africa; shimaa.amin10@yahoo.com (S.A.H.A.M.); sikabwenoki7@gmail.com (S.N.); ramchurane@ukzn.ac.za (E.J.R.); 2 Department of Chemistry, College of Science, King Saud University, P.O. Box 2455, Riyadh 11451, Saudi Arabia; aelfaham@ksu.edu.sa; 3 Chemistry Department, Faculty of Science, Alexandria University, P.O. Box 426, Ibrahimia, Alexandria 12321, Egypt; 4 School of Chemistry and Physics, University of KwaZulu-Natal, Durban 4001, South Africa; 5 Department of Organic Chemistry, University of Barcelona, Barcelona 08028, Spain; 6 CIBER-BBN, Networking Centre on Bioengineering, Biomaterials and Nanomedicine, Barcelona Science Park, Barcelona 08028, Spain; 7 KRISP, College of Health Sciences, University of KwaZulu-Natal, Durban 4001, South Africa

**Keywords:** antimicrobial peptides, teixobactin, lipophilicity, solid-phase peptide synthesis, cyclic depsipeptides

## Abstract

Teixobactin is a recently described antimicrobial peptide that shows high activity against gram-positive bacteria as well as *mycobacterium tuberculosis*. Due to both its structure as a head-to-side chain cyclodepsipeptide and its activity, it has attracted the attention of several research groups. In this regard, a large number of analogs with substitutions in both the cycle and the tail has been described. Here, we report the contribution of the *N*-terminus residue, *N*-Me-d-Phe, to the activity of Arg_10_-teixobactin. On the basis of our findings, we conclude that the *N*-terminus accepts minimum changes but not the presence of long alkyl chains. The presence of a positive charge is a requirement for the activity of the peptide. Furthermore, acylation of the *N*-terminus leads to total loss of activity.

## 1. Introduction

**Figure 1:**
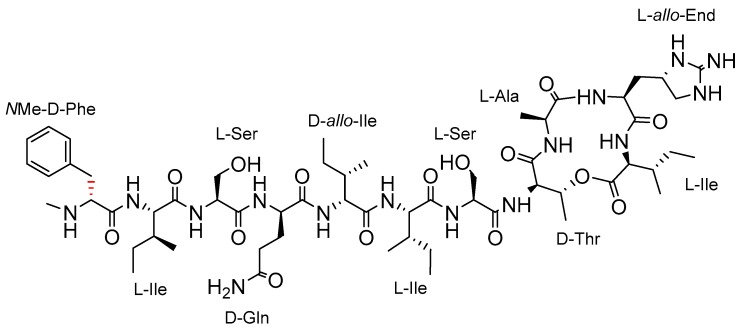
Chemical structure of teixobactin.

The isolation of a new anti-microbial peptide (AMP) called teixobactin in 2015 by Ling et al. raised great expectations because it is one of the few new antibiotics to have been reported in recent years [1,2]. This antibiotic shows high activity against gram-positive bacteria as well as *Mycobacterium tuberculosis*. Teixobactin (Figure 1) is a head-to-side chain cyclodepsipeptide that contains hydrophilic and hydrophobic residues [3,4]. Thus, the tail of the natural peptide contains four polar amino acids, l-*allo*-enduracididine, two Ser, and d-Gln, and four non-polar Ile residues, one Ala and one *N*-Me-d-Phe residue at the *N*-terminus. Finally, a d-Thr is the branching unit to form the cyclic moiety.

Taking Arg_10_-teixobactin (**1**) as a reference, in which the unusual l-*allo*-Enduracididne is replaced by l-Arg [2,5], Nowick’s and our group have independently confirmed the importance of maintaining a balance between the hydrophobic and hydrophilic residues for keeping the activity [3,4]. The main conclusions drawn from those studies are that the replacement of polar or neutral amino acids by Lys retains or improves the activity of the molecule, while replacing the non-polar amino acids by Lys leads to total loss of activity. Only the replacement of non-polar Ile by cyclohexylglycine (Chg) has been reported to retain activity [4]. In a further step towards understanding the contribution of the cationic residues to the activity of teixobactin, our group has prepared a library of more than 25 teixobactin analogs in which extra basic residues (Lys, Orn, Dap) were introduced into positions occupied by polar/neutral amino acids, without affecting the *N-*terminus residue [6]. That study concluded that the activity of a cationic teixobactin analog requires a maximum of three or four positive charges. Thus, the introduction of an extra charge leads to total loss of activity [6].

However, little attention has been paid to the role of the *N*-terminus residue for teixobactin, *N*-Me-d-Phe. We and Madder-Taylor-Singh’s group have demonstrated the importance of the d-configuration, and reported that the Me group cannot be switched for an acetyl (*N*-Ac-d-Phe-Arg_10_-teixobactin (**9**)) [5,7]. Furthermore, in a totally different approach, Nowick et al. prepared a short version of teixobactin, namely lipobactin, which retains most of its activity and in which the last five residues (two Ile (l, and d-allo), Ser, d-Gln, and *N*-Me-d-Phe) are replaced by a dodecanoyl moiety [4,8].

With the aim to further explore the contribution of the *N*-Me-d-Phe to the activity of teixobactin, here we synthesized a small library of Arg_10_-teixobactin analogs. As we previously demonstrated that the replacement of *N*-Me-d-Phe by *N*-Me-d-Lys leads to total loss of activity [6], here we concentrated on increasing the hydrophobicity of the *N*-terminus by introducing extra tails to this terminus. Lipophilicity plays a crucial role in the activity of all peptides, as well as in their transport and distribution within biological systems [9,10,11]. However, for AMPs, increasing hydrophobicity can be a way to promote binding to bacterial membranes, thereby causing greater disruption. For instance, some species such as *S. aurus* show a limited electrostatic surface potential with the hydrophilic AMPs, causing the AMPs to bind to the bacterial membrane in a different way [12]. Furthermore, the membrane binding of such peptides is salt sensitive, leading to limited potency at physiological ionic strength; however, increasing their hydrophobicity means reducing their salt sensitivity, as well as increasing their bactericidal potency. As an example, increasing the hydrophobicity of the AMP CNY21 (CNYITELeRRQHARASHLGLAR) improved peptide adsorption, led to peptide liposome leakage, and enhanced activity against *Escherichia coli* and *Pseudomonas aeruginosa* [12]. Furthermore, it has been demonstrated that the replacement of the aliphatic acyl moiety of polymyxin B or colistin by aromatic or fatty acid substituents has a great impact on the antimicrobial activity of the analogs [13,14,15,16,17].

## 2. Results and Discussion

### 2.1. Synthetic Strategy

**Figure 2:**
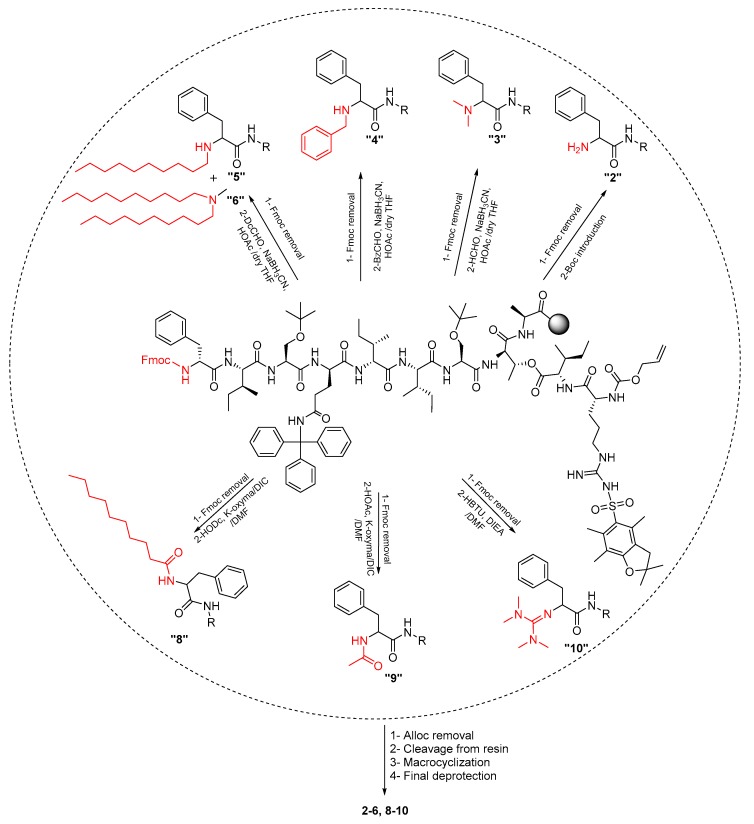
Chemical structure of the teixobactin analogs **2**–**6**, **8**–**10** used in this study; “**2**–**6**, **8**–**10**” are the protected linear precursors of **2**–**6**, **8**–**10**.

The *N*-terminus Arg_10_-teixobactin analogs (Figure 2, **2**–**6**, **8**–**10**) were synthesized using strategies developed by our group for the previous analogs [2,3,7].

Briefly, chloro-tritylchloride (CTC) polystyrene resin was used for the elongation of the peptide chain using Fmoc-amino acids, with Ala as C-terminus, using 1-[bis-(dimethylamino)methylene]-1*H*-1,2,3-triazolo[4,5-*b*]-pyridinium 3-oxide hexafluorophosphate (HATU)/Diisopropylethylamine (DIEA) as coupling method and dimethylformamide (DMF) as solvent. After the incorporation of Fmoc-Ile_6_, acylation of the β-hydroxyl group of d-Thr was carried out with Alloc-Ile-OH/DIC-DMAP in DMF-dicholoromethane (DCM) (8:2), followed by the removal of the Alloc group, and further acylation with Alloc-Arg (Pbf)-OH. The introduction of the rest of the amino acids was then continued using Fmoc chemistry. The last amino acid, d-Phe, was incorporated as an Fmoc derivative. In our previous synthesis, the last residue was Boc-*N*-Me-d-Phe, with the aim to protect the *N-*terminus during cyclization [2,3,6,7].

Analog **2**, which has a free amine at *N*-terminus, was synthesized by removing the Fmoc of d-Phe and then reprotecting the amino function as Boc by means of Boc_2_O/DIEA (1:2) in DMF, to prevent its reaction during the cyclization step.

The remaining modifications at the *N*-terminus were performed in solid-phase through alkylation, acylation, or guanidination, after the removal of the last Fmoc group. The first group (**3**–**6**) was achieved by the reductive amination of the d-Phe analog by reaction with the corresponding aldehydes in the presence of NaBH_3_CN and HOAc in dry tetrahydrofuran (THF). The reaction with formaldehyde-led, as expected, to a complete double methylation (**3**). The reaction with decanal was carried out with excess aldehyde to trigger the formation of an approximately equimolar mixture of the mono- (**5**) and the di- (**6**) decanyl derivatives. These compounds were separated after the cyclization step, which was carried out in solution, and subjected to deprotection. Finally, the reaction with benzaldehyde rendered only the mono derivative (**4**). Acylation with decanoic acid of the d-Phe analog took place smoothly with DIC and K-Oxyma, rendering the decanoyl derivative (**8**).

**Scheme 1:**
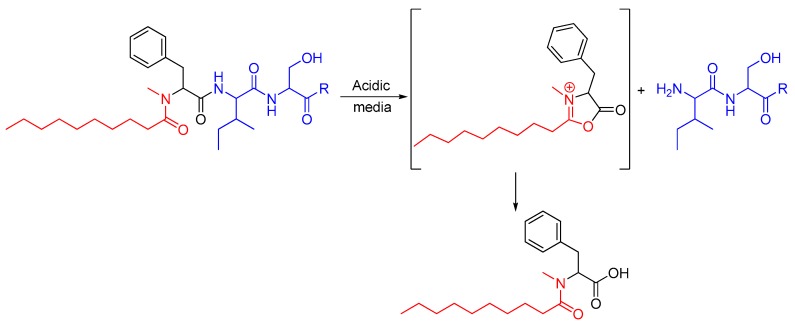
Loss of *N*-Dec-*N*-Me-d-Phe terminus of analog **7** through oxazolonium formation.

Of note, an attempt to acylate *N*-Me-d-Phe with decanoic acid failed (**7**). Although the coupling of decanoic acid took place efficiently, acidic cleavage with 2% trifluoroacetic acid (TFA) rendered a compound with a mass corresponding to a loss of *N*-Dec-*N-*Me-d-Phe. This kind of fragmentation has already been described in the literature [18], and occurs as a result of the formation of oxazolonium ion as an intra-reaction rearrangement (Scheme 1). Since this reaction was reported when acetylation was attempted, we were confident that the longer chain of the decanoyl vs. the acetyl would minimize the formation of the side reaction, thereby allowing us to obtain small amounts of the target analog. Unfortunately, our hypothesis failed and the compound was not obtained. However, our findings demonstrate that this is a general reaction for the *N*-acyl *N*-methyl peptides.

Finally, the modification of the *N-*terminus to obtain the tetramethyl-*N*-guanidino-d-Phe (**10**) was achieved by treating the free d-Phe *N*-terminus with HBTU in the presence of DIEA in DMF for 3 h [19].

For all the derivatives except **2**, the Alloc protecting group of Arg was removed and all partially protected peptides were cleaved from the resin using 2% TFA in DCM, without re-protection of the secondary amines **4** and **5**. Cyclization of the analogs was carried out in solution with PyAOP and OxymaPure in DMF–DCM (1:9) for 2 h. Finally, global deprotection was performed with TFA–TIS–H_2_O (95:2.5:2.5) for 2 h. All peptides except **8** were purified by semi-preparative HPLC before testing their biological activity.

The most hydrophobic analogs, **5**, **6**, and **8**, presented several difficulties regarding purification. In this regard, it was not possible to dissolve compound **8** in any solvent system compatible with reverse-phase chromatography. Nevertheless, by washing the solid obtained several times with 30% acetonitrile in water, impurities from the cyclization were removed, as were the protecting groups, and the decanoyl derivative was obtained in good purity (Figure S11). The mixture of compounds **5** and **6** was also a challenge with respect to solubilization. In this case, the addition first of 100 μL of TFA and then 7 mL of acetonitrile, followed by sonication for 15 min, yielded a clear solution. Then, 3 mL of water was added slowly and no precipitation of the compounds was observed. This solution was injected into the semi-preparative HPLC, and the two analogs were separated efficiently.

### 2.2. Microbiological Evaluation

After the purification and characterization of the new analogs by MALDI-TOF, their antimicrobial activity was tested against two gram-positive bacteria strains, *Staphylococcus aureus* and *Bacillo subtilis,* as well as two gram-negative strains, namely *Escherichia coli* and *Pseudomonas aeruginosa* (Table 1). The compounds were dissolved at a concentration of 6 mg/mL in 10% DMSO for compounds **1**–**4** and 60% DMSO for compounds **5**, **6**, and **8** because of their poor solubility in water. They were then diluted to reach 512 μg/mL, the highest concentration tested, followed by two-fold serial dilution. To ensure that DMSO did not interfere in the results, a blank solution of 60% DMSO under the same dilutions was also assayed.

The Minimum Inhibitory Concentration (MIC) results in Table 1 indicate that an increase in the lipophilicity of the alkyl substituents leads to a decrease in activity. Thus, the best activity was found for the parent compound (**1**), followed by the analog without any substitution (**2**). The dimethyl derivative (**3**) and the benzyl one (**4**) retained some activity. On the other hand, the two analogs with one and two decanyl substituents (**5**, **6**) showed total loss of activity. Acylation with decanoic acid (**8**) followed the same trend as with the long alkyl chain (**5**) or just with Ac (**9**). Although the positive charge was found to be a requirement for the activity (**1**, **2**, **3**, **4**), the tetramethylguanidino analog (**10**) also showed a significant loss of activity. These data indicate that the modification of the *N-*terminus of teixobactin is critical for the activity of the peptide [20].

## 3. Materials and Methods

### 3.1. Materials

All reagents and solvents were purchased from commercial sources. Amino acids and 2-chlorotrityl-chloride (CTC) resin were obtained from (Iris Biotech, Marktredwitz, Germany) and *N*,*N*-Diisopropylethylamine (DIEA) from (Sigma-Aldrich, St. Louis, MO, USA). *N,N,N′,N′-*tetramethyl-*O*-(1*H*-benzotriazol-1-yl)uronium hexafluorophosphate (HBTU) and 1-cyano-2-ethoxy-2-oxoethyliden(aminooxy) dimethylamino-morpholino-carbenium hexafluoro phosphate (COMU) were a gift from Luxembourg Technologies. Organic solvents were supplied by (Merck, Darmstadt, Germany).

### 3.2. Instruments

Analytical HPLC was performed on an Agilent 1100 system using a Phenomex LunaC_18_ (3 μm, 4.6 mm × 50 mm) column (Agilent, Santa Clara, CA, USA), with different gradient methods and using Buffer A: 0.1% TFA in H_2_O; buffer B: 0.1% TFA in CH_3_CN as mobile phase. A flow rate of 1.0 mL/min and UV detection wavelength of 220 nm were used.

Crude peptides were purified on a Shimadzu LC-8A semi-preparative HPLC using a Phenomenex LunaC_18_ (2) column (10 μm, 10 mm × 250 mm), with a flow rate of 7.0 mL/min. The wavelength detection and mobile phase gradients were the same as those employed for analytical HPLC. Mass spectrometry was carried out using a Bruker Autoflex III MALDI-TOF mass spectrometer (Bruker, Billerica, MA, USA) in positive mode and α-Cyano-4-hydroxycinnamic acid (ACH) as the matrix.

### 3.3. Methods

#### 3.3.1. Chemistry

All Solid Phase Peptide Synthesis (SPPS) steps were carried out manually in a 10-mL polypropylene syringe fitted with polypropylene frits at room temperature. CTC resin (166 mg, 1.69 mmol/g) was activated using SOCl_2_–DCM (1:9) (10 mL) overnight. The first amino acid, Fmoc-l-Ala-OH (31 mg, 0.1 mmol, 1 eq.), was loaded onto the CTC resin in DCM (0.5 mL) in the presence of DIEA (174 µL, 1 mmol, 10 eq.) as a base and was shaken for 1 h. MeOH (100 µL) was then added to the reaction mixture, which was left to shake for an additional 30 min to ensure the total capping of free active sites in the CTC resin.

The rest of the amino acids were incorporated as follows: Fmoc-AA-OH (0.3 mmol, 3 eq.)/HATU (114 mg, 0.3 mmol, 3 eq.)/DIEA (104 µL, 0.6 mmol, 6 eq.) (3:3:6) in DMF (0.5 mL) for 30 min. Fmoc removal was accomplished with 20% piperidine in DMF (2 × 2 mL, 5 min). The resin was washed with DMF (2 × 5 mL), DCM (2 × 5 mL), and DMF (2 × 5 mL) between steps. Esterification was performed after coupling Fmoc-Ile_6_-OH using Alloc-l-Ile_11_-OH (215 mg, 1 mmol, 10 eq.), DIC (154 µL, 1mmol, 10 eq.) and DMAP (12 mg, 0.1 mmol, 1 eq.) in DCM–DMF (8:2) (0.5 mL) for 2 h.

The Alloc group was removed under nitrogen in a vial shielded from light. The peptide was treated with a mixture of tetrakistriphenylphosphine palladium (0) (11 mg, 0.01 mmol, 0.1 eq.) and phenylsilane (124 µL, 1 mmol, 10 eq.) in dry DCM (1 mL) for 15 min. The peptide resin was then washed with DMF (2 × 5 mL), DCM (2 × 5 mL), and DMF (2 × 5 mL).

Reductive amination was carried out using formaldehyde, benzaldehyde, or decanal (0.6 mmol, 6 eq.) in the presence of NaBH_3_CN (1.6 mmol, 16 eq.) and AcOH (120 µL) in dry THF (0.5 mL) for 2 h. Coupling with decanoic acid (52 mg, 0.3 mmol, 3 eq.) was performed with DIC (46 µL, 0.3 mmol, 3 eq.) and K-Oxyma (54 mg, 0.3 mmol, 3 eq.) in DMF for 2 h. Guanidine formation was achieved using HBTU (113 mg, 0.3 mmol, 3 eq.) in DMF (0.5 mL) in the presence of DIEA (104 µL, 0.6 mmol, 6 eq.). Boc protection was achieved by treating the unprotected peptide with Boc_2_O (218 mg, 1 mmol, 10 eq.) and DIEA (174 μL, 1 mmol, 10 eq.) in DMF (2 mL) for 2 h.

The partially protected peptide was cleaved from the CTC resin with TFA–DCM (2:98) (5 mL) for 10 min, and the solution was filtered off. The treatment was repeated two more times and the combined filtrates were evaporated to dryness.

Cyclization of all peptides was done in solution by dissolving the corresponding protected peptide (≈0.1 mmol) in DMF (1 mL), followed by the addition of a mixture of DIEA (0.6 mmol, 6 eq.) and OxymaPure (0.3 mmol, 3 eq.) in DCM (50 mL). Extra DCM was then added to reach a concentration of 0.1 mM. The reaction mixture was cooled to 0 °C and PyAOP (0.3 mmol, 3 eq.) was added. After 24 h, DCM was evaporated, and DMF was removed by freeze-drying.

Global deprotection was achieved by treating the fully protected peptide TFA–TIS–H_2_O (95:2.5:2.5) (5 mL) for 4 h, followed by washing with cold ether till the peptide precipitated. The supernatant was then discarded, and the washing was repeated three more times. The solid residue was then dried. All peptides except analog **8** were purified by semi-preparative HPLC. In each case, fractions containing the expected peptide were combined, lyophilized, characterized by MALDI-TOF, and submitted to antimicrobial testing.

#### 3.3.2. Determination of Minimum Inhibitory Concentration (MIC)

The MIC was determined using the broth micro dilution method, as described by the Clinical and Laboratory Standards Institute (CLSI) guidelines, using two gram-positive and two gram-negative bacteria strains (*Staphylococcus aureus* ATCC 29213, *Bacillus subtilis* ATCC 6051, *Escherichia coli* ATCC 25922, *Pseudomonas aeruginosa* ATCC 27853). First, the bacterial strains were sub-cultured onto Mueller Hinton agar and incubated at 37 °C for 24 h before use in the experiments. Stock solutions at 6 mg/mL in 10% DMSO were prepared for analogs **1**–**4** and 60% DMSO for analogs **5**, **6**, and **8**. Second, two-fold serial dilutions of each drug/compound were made in cation-adjusted Mueller Hinton broth (CAMHB) in a 96-well microtiter plate. The bacterial inoculum was prepared in distilled water, matched to a 0.5 McFarland standard, and added to make a final volume of 200 µL in each microliter well. The plates were incubated for 24 h at 37 °C under aerobic conditions. The MIC was then recorded as the lowest concentration at which no visible growth occurred. Standard drug, media, and bacterial growth control wells were included in each plate. Meropenem was used as a positive control and a blank solution of 60% DMSO in water was also tested to ensure that solvent did not affect the bacterial strains. The assay was performed in triplicate to confirm the results.

## 4. Conclusions

In conclusion, the *N-*terminus part of teixobactin accepts minimum changes. Analogs with activity were achieved only when the Me group from d-Phe was absent or substituted by Bzl, or an extra Me was present. Interestingly, all analogs that contained a fatty chain either in the form of an acyl or alkyl showed total loss of activity. The lack of activity of analog **8**—where the *N-*terminal was acylated with decanoic acid—could be understandable, because in this analog the positive charged was masked as happened in the acyl analog **9**, which was not active either. On the other hand, the lack of activity of analogs **5** and **6**, which contained one and two decanyl chains, respectively, was more unexpected. In this sense, it should be taken into account that several peptides such as CNY21 enhanced their activity when lipophilicity was added [12]. Furthermore, the tail of the head to side-chain cyclo peptide (similar topology than teixobactin) of polymyxin B or colistin ended with a fatty acid. To explain the lack of activity of analogs **5** and **6**, it can be speculated that lipophilicity in cationic peptides such as CNY21 or polymyxin B/colistin translates to an increase of activity. On the other hand, the same lipophilicity on already lipophilic peptides such teixobactin could provoke a loss of activity due to the creation of lipophilic interactions between the fatty chain and the peptide.

## Figures and Tables

**Table 1:** Minimum Inhibitory Concentration (MIC) (µg/mL) for teixobactin analogs (**1**–**6**, **8**–**10**).

		Gram+		Gram−	
		*S. aureus* ATCC 29213	*B. subtilis* ATCC 6051	*E. coli* ATCC 25922	*P. aerug* ATCC 27853
	Teixobactin ^1^	0.25	0.06	25	>32
**1**	*N*-Me-d-Phe-Arg_10_-teixobactin	2	0.5	64	NI ^1^
**2**	d-Phe-Arg_10_-teixobactin	2	1	32	NI
**3**	*N*-Me_2_-d-Phe-Arg_10_-teixobactin	16	2	128	NI
**4**	*N*-Bz-d-Phe-Arg_10_-teixobactin	8	2	NI	NI
**5**	*N*-De-d-Phe-Arg_10_-teixobactin ^1^	NI	128	NI	NI
**6**	*N*-De_2_-d-Phe-Arg_10_-teixobactin	NI	128	NI	NI
**8**	*N*-Dec-d-Phe-Arg_10_-teixobactin ^1^	NI	NI	NI	NI
**9**	*N*-Ac-d-Phe-Arg_10_-teixobactin ^2^	NI	NI	NI	NI
**10**	*N*-Tmg-d-Phe-Arg_10_-teixobactin ^1^	256	64	NI	NI

^1^ De: decanyl, Dec: decanoyl, Tmg: tetramethylguanidino, NI: No inhibition, ^2^ Described earlier by our group [7].

## Supplementary Materials

The supplementary materials are available online.
