# Supplementary material for: Investigation of the N-Terminus Amino Function of Arg10-Teixobactin
Source: Molecules. 2017 Sep 28;22(10):1632. doi: 10.3390/molecules22101632 (PMC6151525; doi:10.3390/molecules22101632)

Supplementary Information (SI)

Investigating of *N*-terminal amino function of Arg$_{10}$-teixobactin

Shimaa A. H. Abdel Monaim[1], Sikabwe Noki[1], Estelle J. Ramchuran[1], Ayman El-Faham[2,3], Fernando Albericio[1,2,4,5,6,*] and Beatriz G. de la Torre[1,7,*]

[1] Catalysis and Peptide Research Unit, School of Health Sciences, University of KwaZulu-Natal, Durban 4001, South Africa; shimaa.amin10@yahoo.com, sikabwenoki7@gmail.com, ramchurane@ukzn.ac.za
[2] Department of Chemistry, College of Science, King Saud University, P.O. Box 2455, Riyadh 11451, Saudi Arabia; aelfaham@KSU.EDU.SA
[3] Chemistry Department, Faculty of Science, Alexandria University, P.O. Box 426, Ibrahimia, Alexandria 12321, Egypt;
[4] School of Chemistry and Physics, University of KwaZulu-Natal, Durban 4001, South Africa; albericio@ukzn.ac.za
[5] Department of Organic Chemistry, University of Barcelona, Barcelona 08028, Spain; albericio@ub.edu
[6] CIBER-BBN, Networking Centre on Bioengineering, Biomaterials and Nanomedicine, Barcelona Science Park, Barcelona 08028, Spain
[7] School of Laboratory of Medicine and Medical Sciences, University of KwaZulu-Natal, Durban 4001, South Africa; garciadelatorreb@ukzn.ac.za
* Correspondence: albericio@ukzn.ac.za; garciadelatorreb@ukzn.ac.za; Tel.: +27-614-009-144

Table of content

Figure S1: HPLC of analogue **2**	S2
Figure S2: MALDI-TOF spectra of analogue **2**	S2
Figure S3: HPLC of analogue **3**	S3
Figure S4: MALDI-TOF spectra of analogue **3**	S3
Figure S5: HPLC of analogue **4**	S4
Figure S6: MALDI-TOF spectra of analogue **4**	S4
Figure S7: HPLC of analogue **5**	S5
Figure S8: MALDI-TOF spectra of analogue **5**	S5
Figure S9: HPLC of analogue **6**	S6
Figure S10: MALDI-TOF spectra of analogue **6**	S6
Figure S11: HPLC of analogue **8**	S7
Figure S12: MALDI-TOF spectra of analogue **8**	S7
Figure S13: HPLC of analogue **9**	S8
Figure S14: MALDI-TOF spectra of analogue **9**	S8
Figure S15: HPLC of analogue **10**	S9
Figure S16: MALDI-TOF spectra of analogue **10**	S9

Figure S1: HPLC of analogue**2** (method 10-60% B in 15 min)

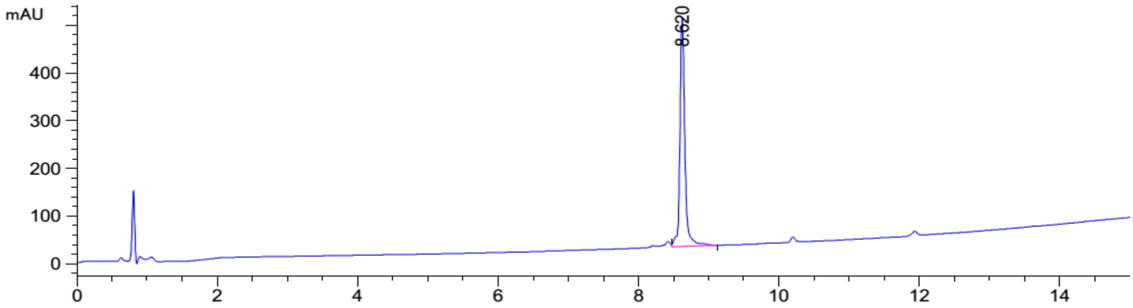

Figure S2: MALDI-TOF spectra of analogue **2** (Expected mass = 1230.4770, observed mass for M+H$^+$, M+Na$^+$, and M+K$^+$)

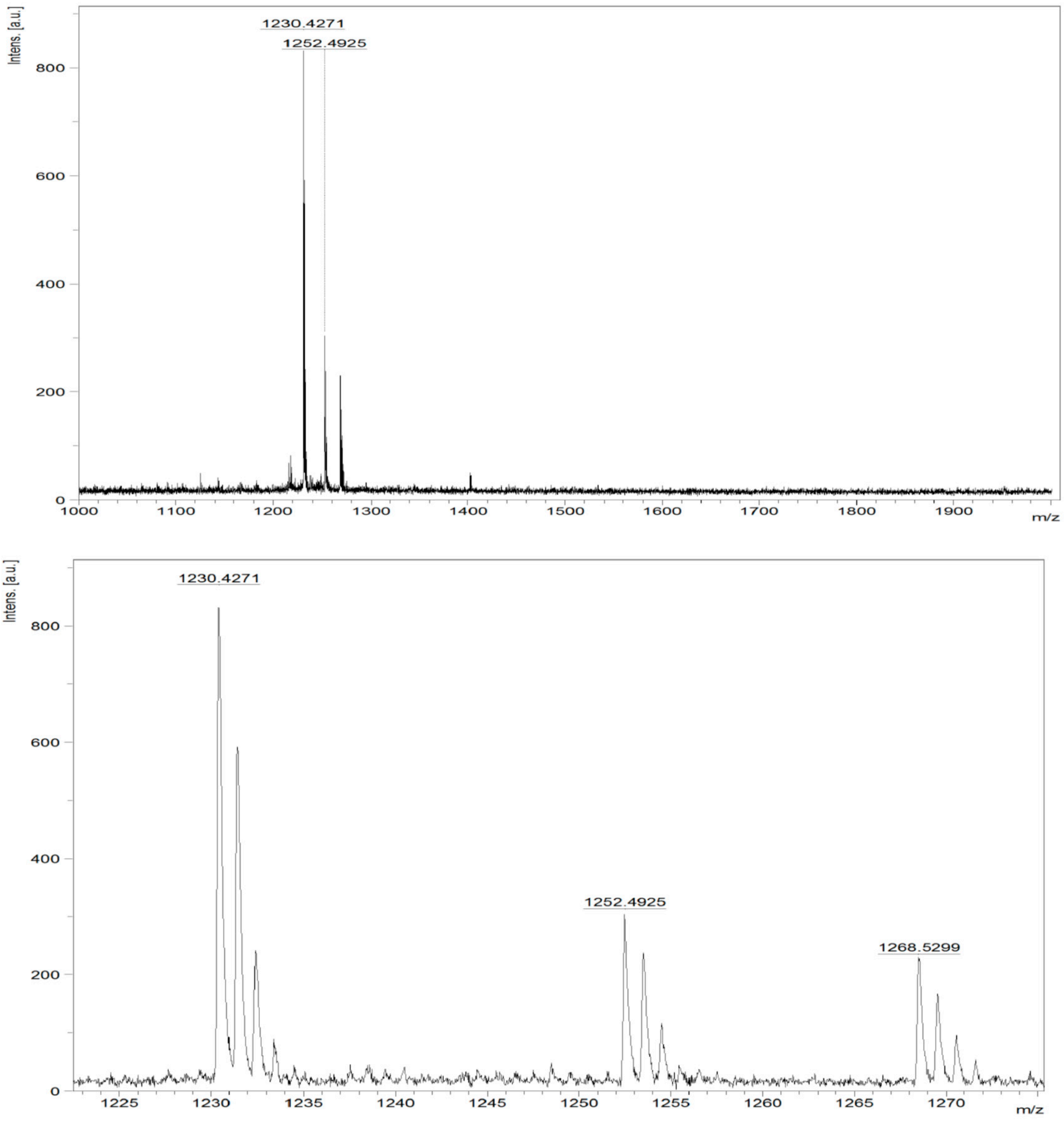

Figure S3: HPLC of analogue**3** (method 25-50% B in 15 min)

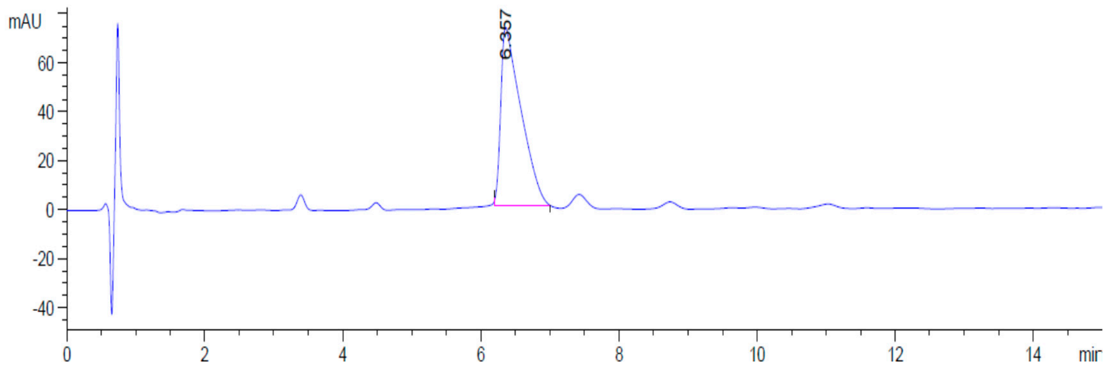

Figure S4: MALDI-TOF spectra of analogue **3** (Expected mass = 1258.5310, observed mass for M+H$^+$, and M+Na$^+$)

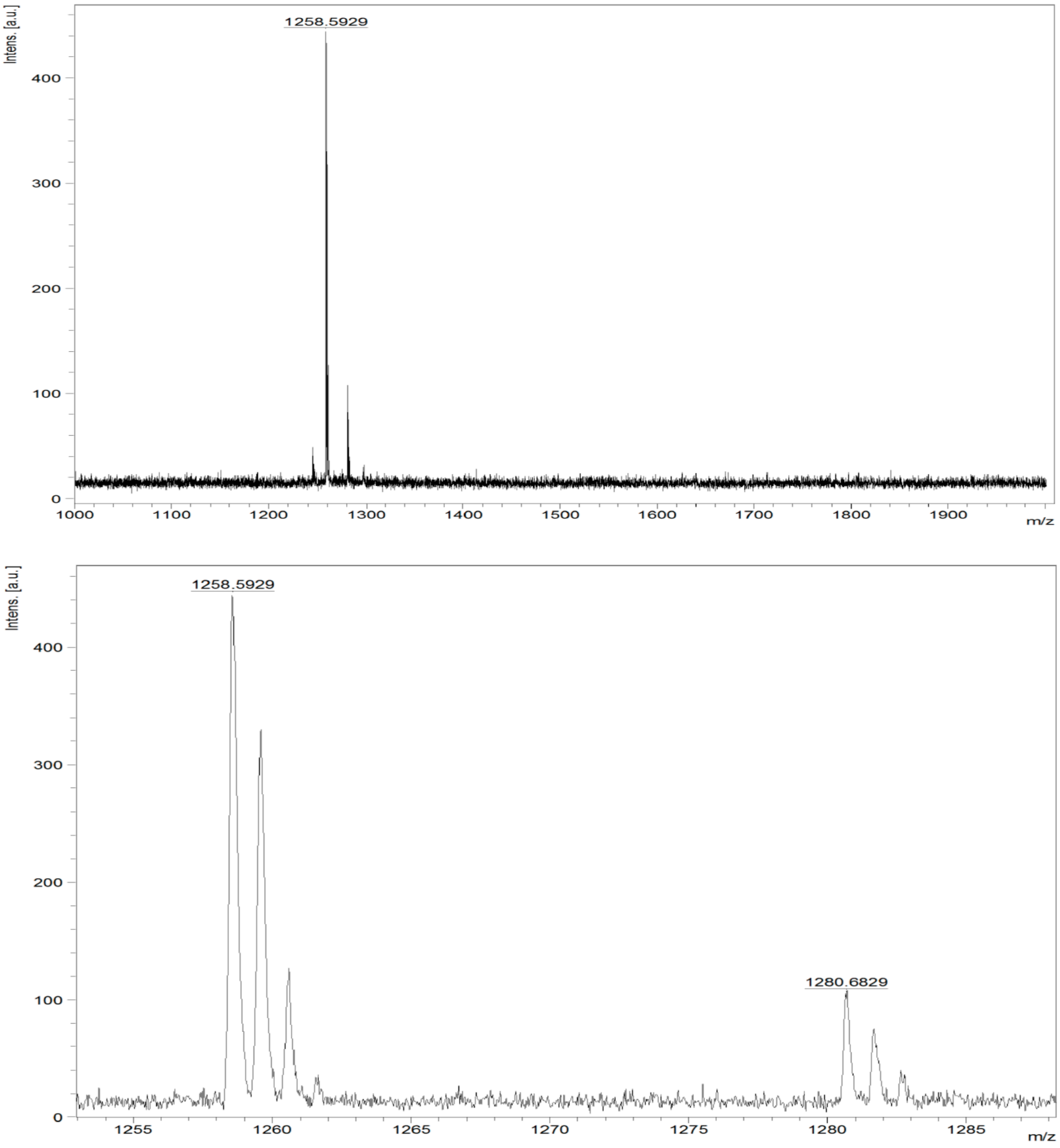

Figure S5: HPLC of analogue **4** (method 25-40% B in 15 min)

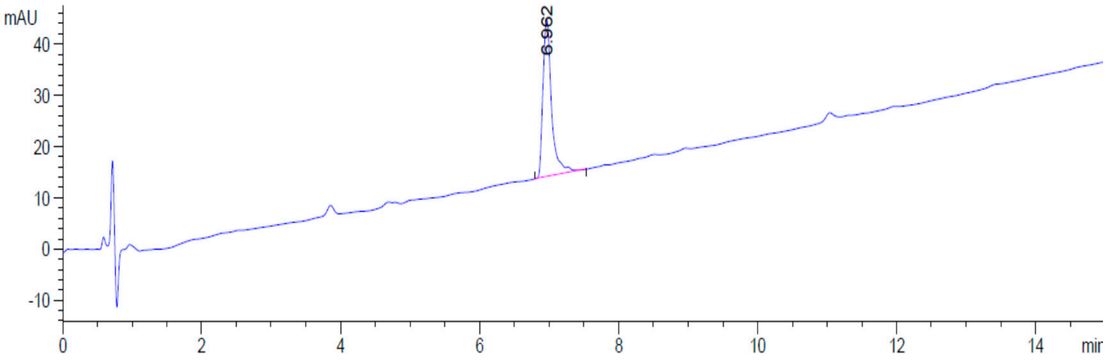

Figure S6: MALDI-TOF spectra of analogue **4** (Expected mass = 1320.6020, observed mass for M+H$^+$, M+Na$^+$, and M+K$^+$)

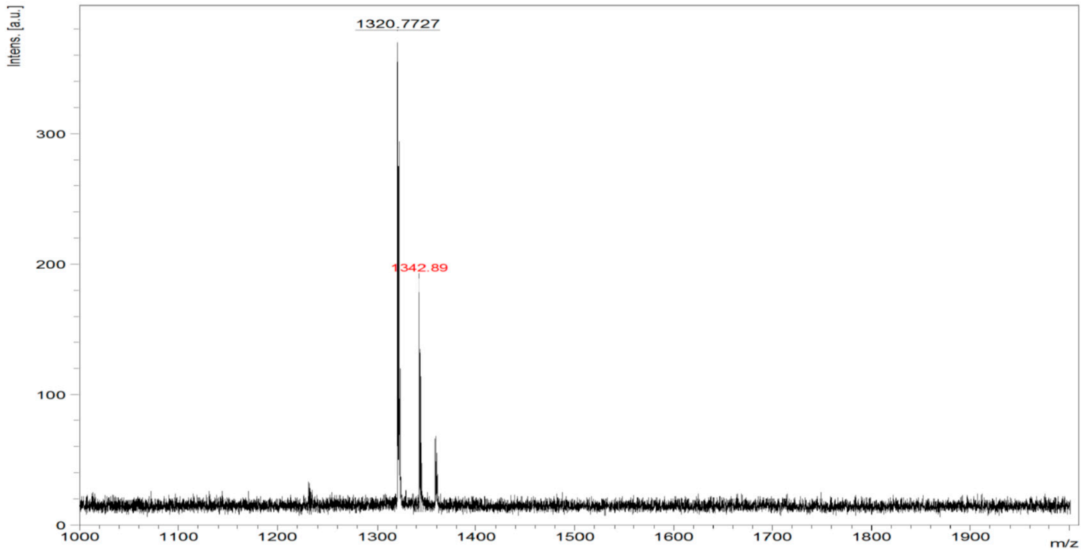

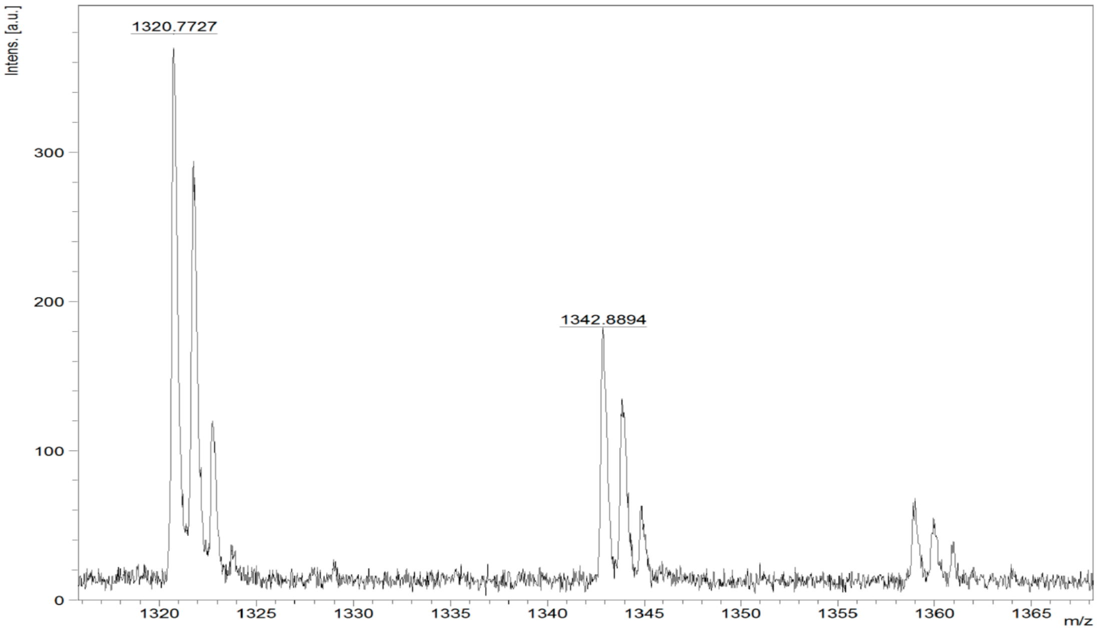

Figure S7: HPLC of analogue **5** (method 10-65% B in 15 min)

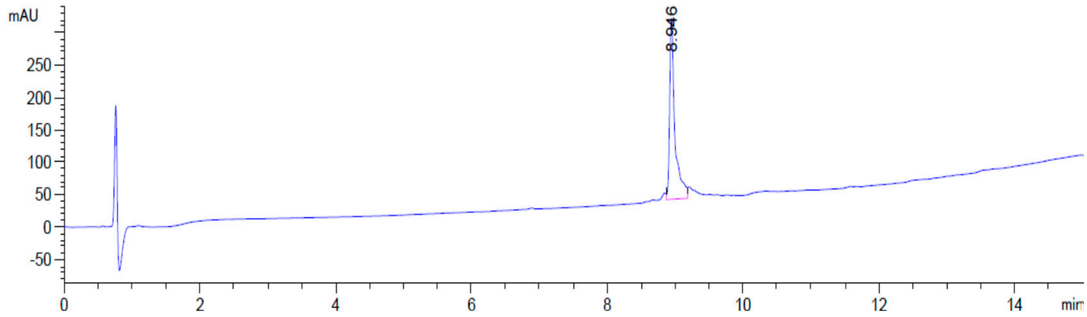

Figure S8: MALDI-TOF spectra of analogue **5** (Expected mass = 1370.7470, observed mass for $M+H^+$, $M+Na^+$, and $M+K^+$)

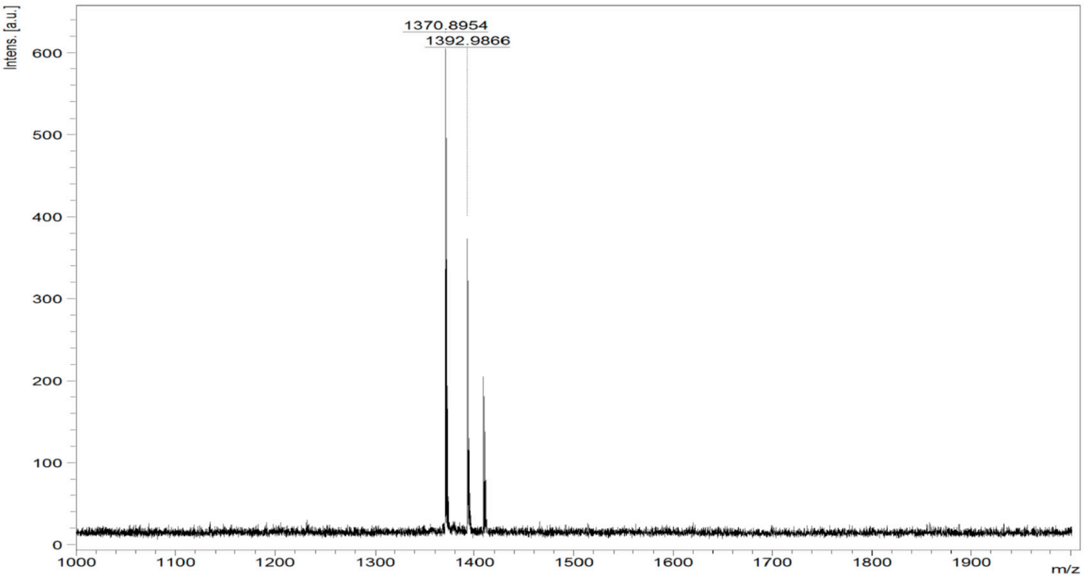

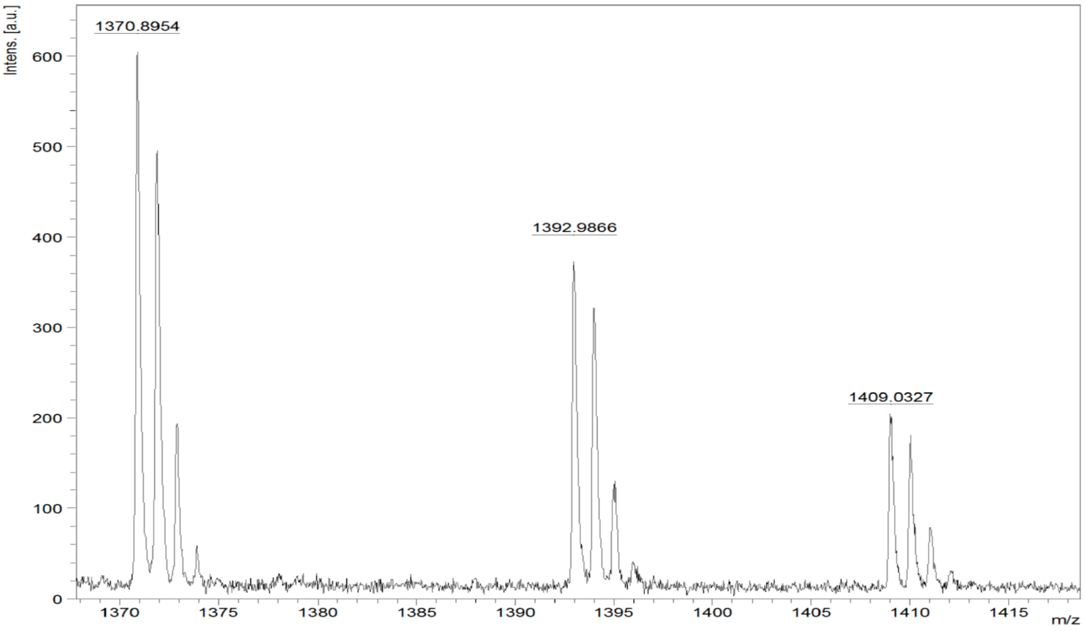

Figure S9: HPLC of analogue **6** (method 10-65% B in 15 min)

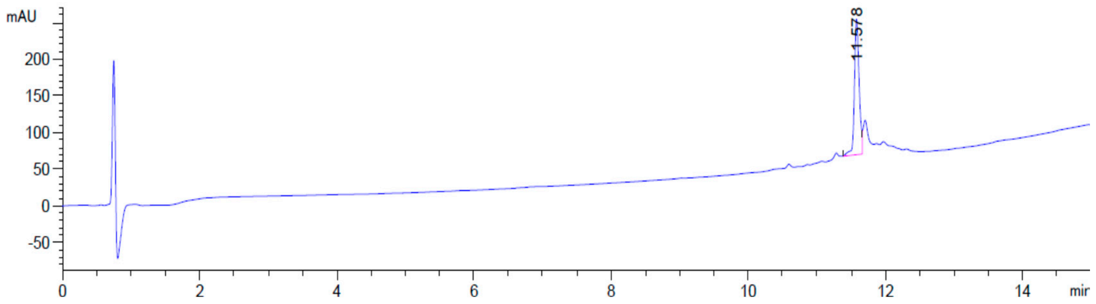

Figure S10: MALDI-TOF spectra of analogue **6** (Expected mass = 1511.0170, observed mass for $M+H^+$, $M+Na^+$, and $M+K^+$)

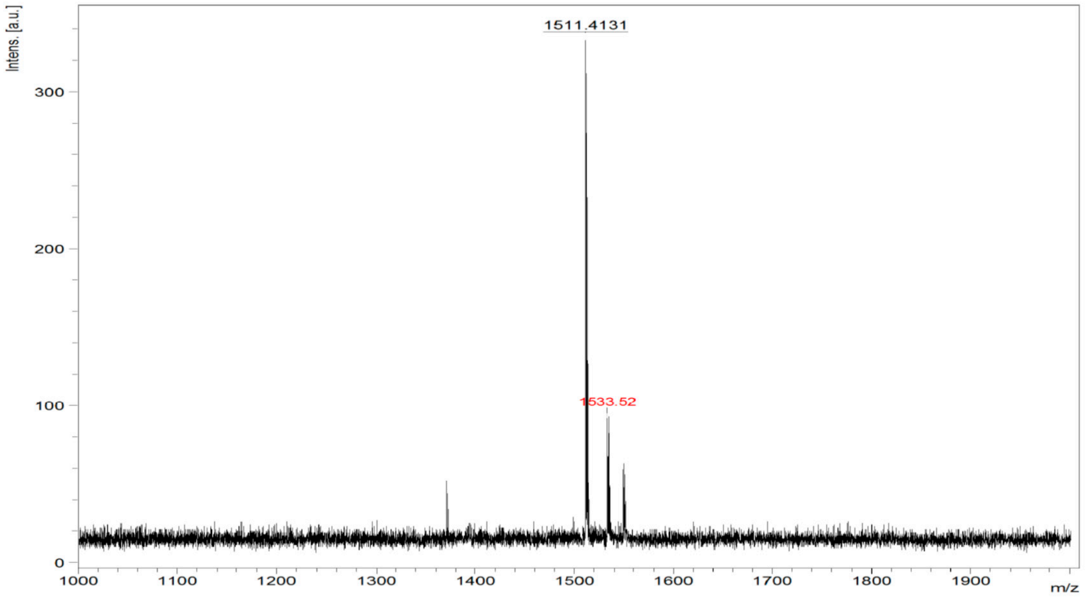

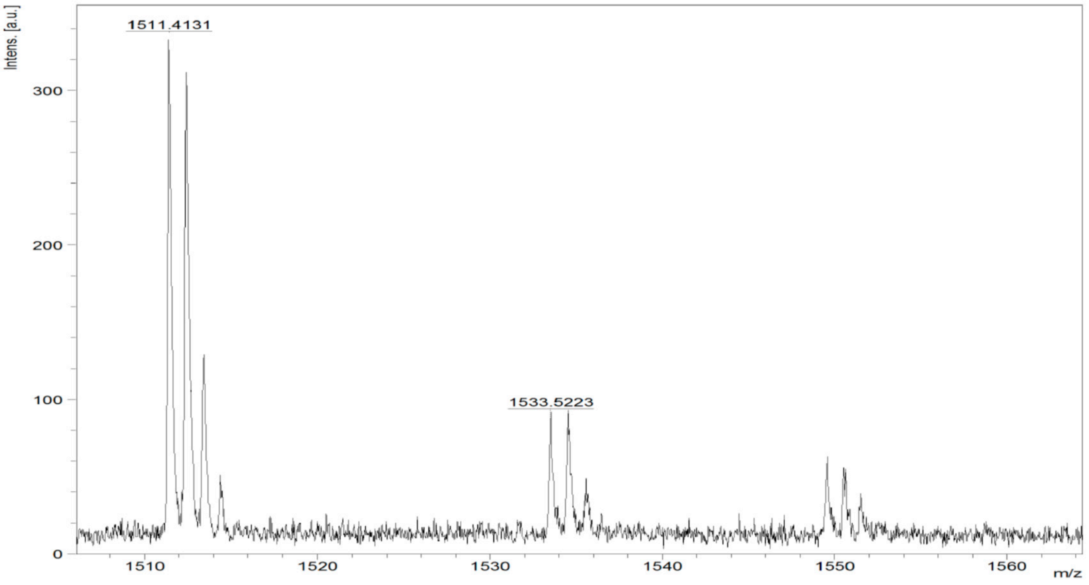

Figure S11: HPLC of analogue **8** (method 45-85% B in 15 min)

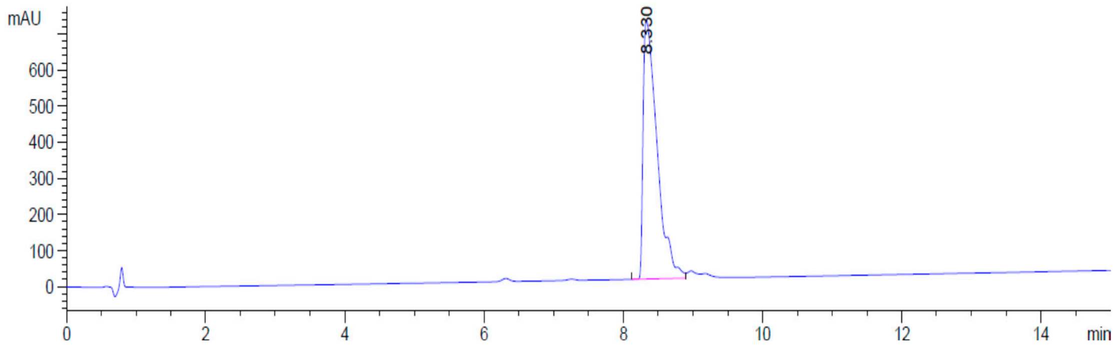

Figure S12: MALDI-TOF spectra of analogue **8** (Expected mass = 1384.7300, observed mass for $M+H^{+}$)

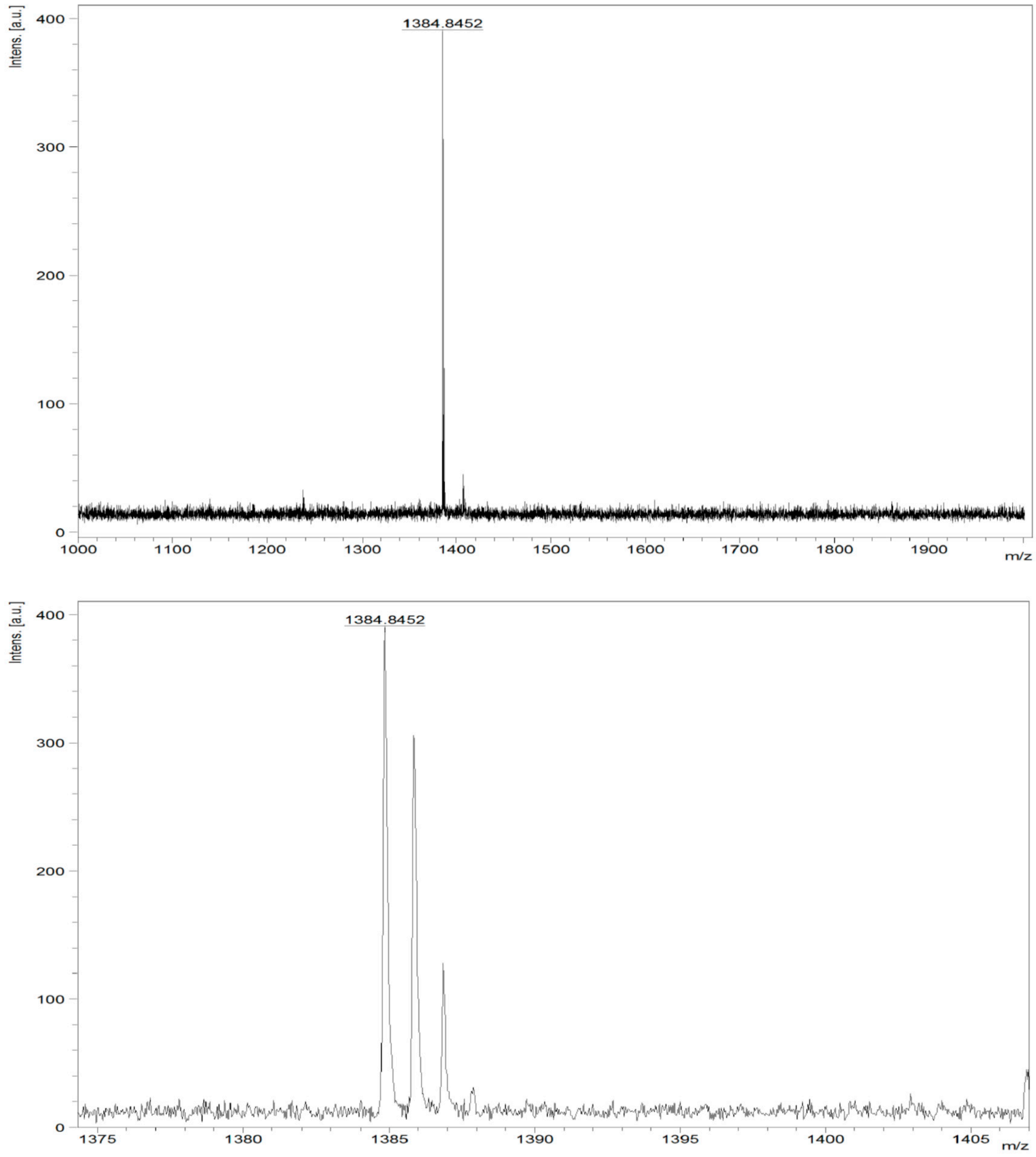

Figure S13: HPLC of analogue **9** (method 30-50% B in 15 min)

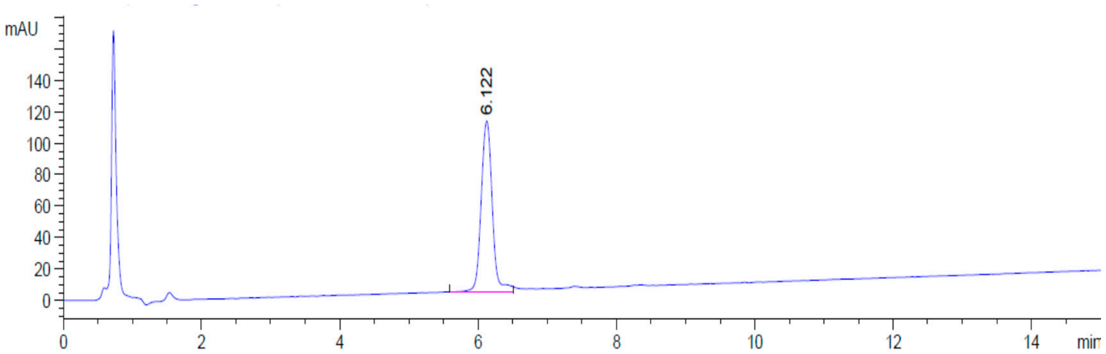

Figure S14: MALDI-TOF spectra of analogue **9** (Expected mass = 1272.5140, observed mass for $M+H^+$)

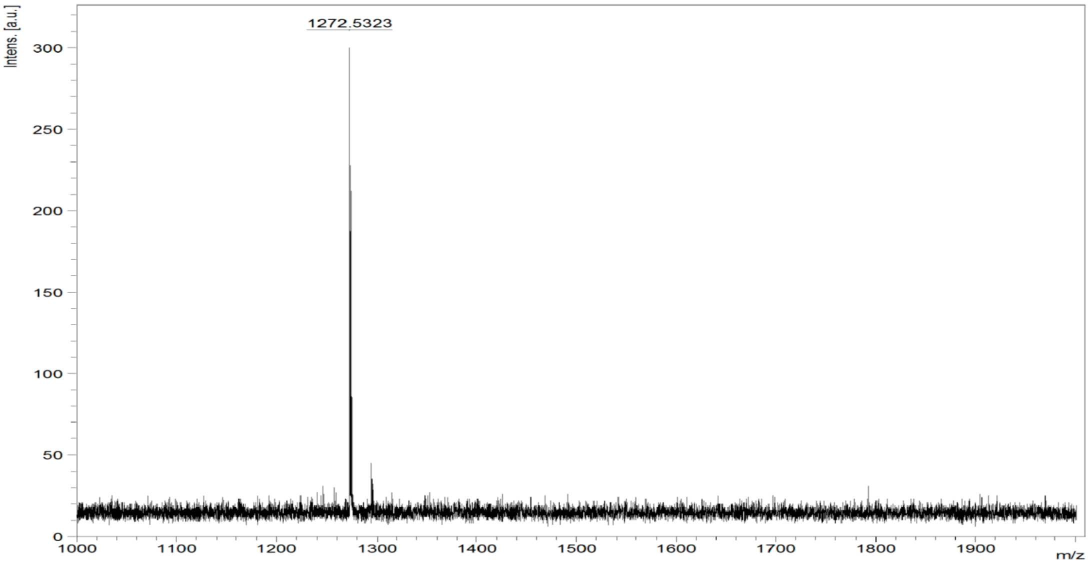

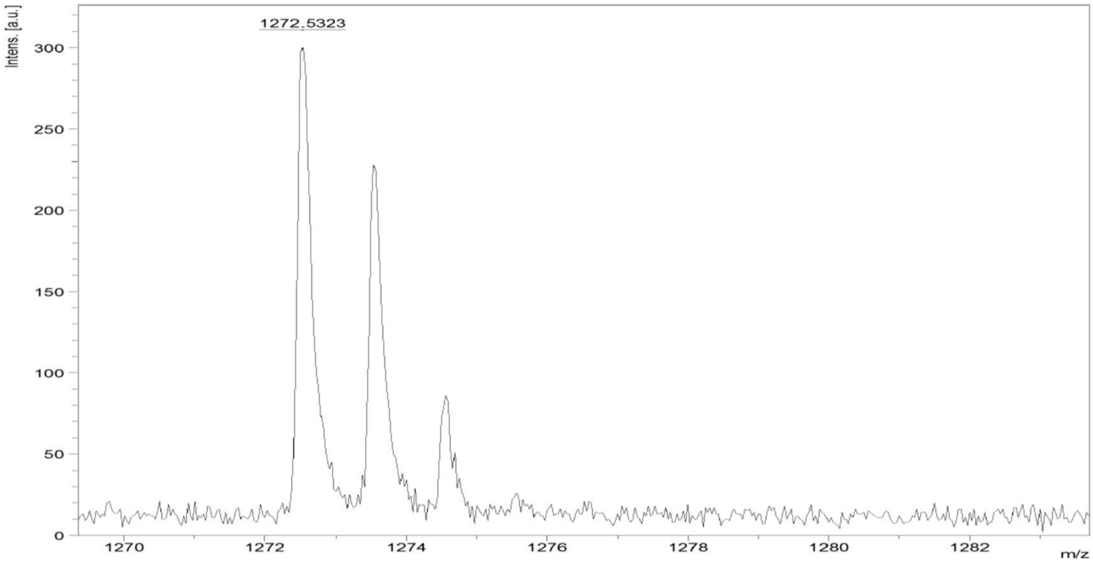

Figure S15: HPLC of analogue **10** (method 30-50% B in 15 min)

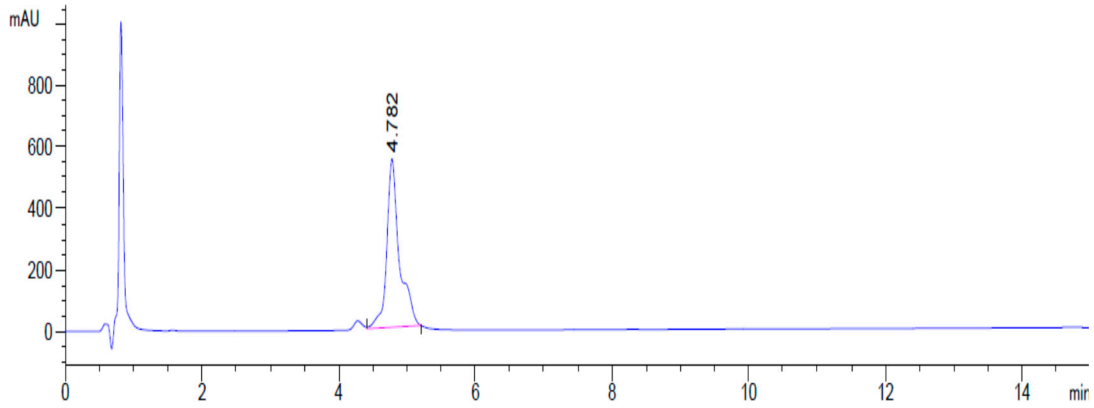

Figure S16: MALDI-TOF spectra of analogue **10** (Expected mass = 1328.6260, observed mass for M+H$^+$, M+Na$^+$, and M+K$^+$)

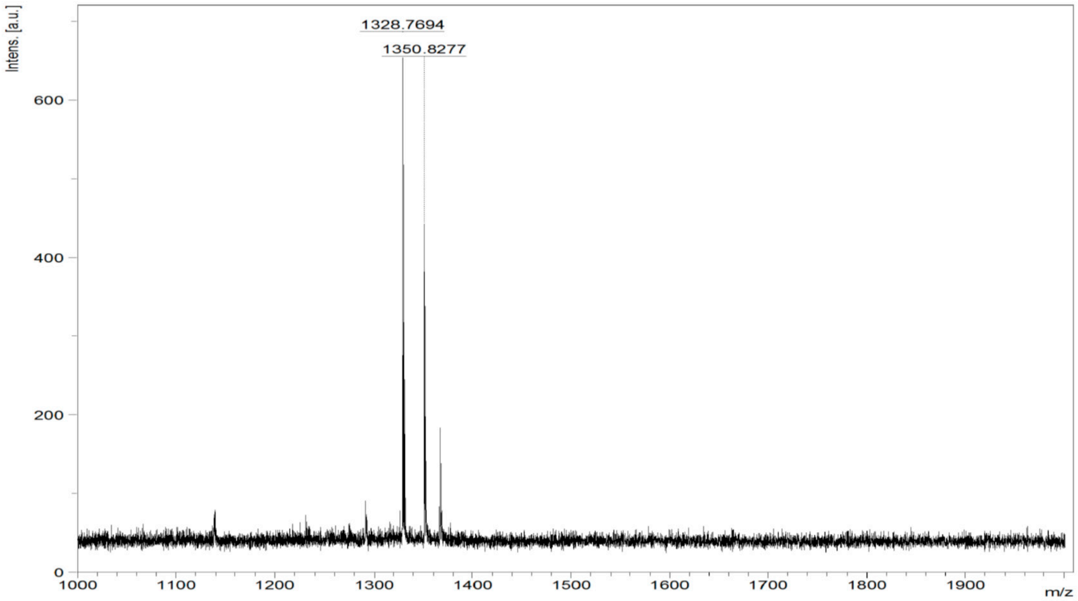

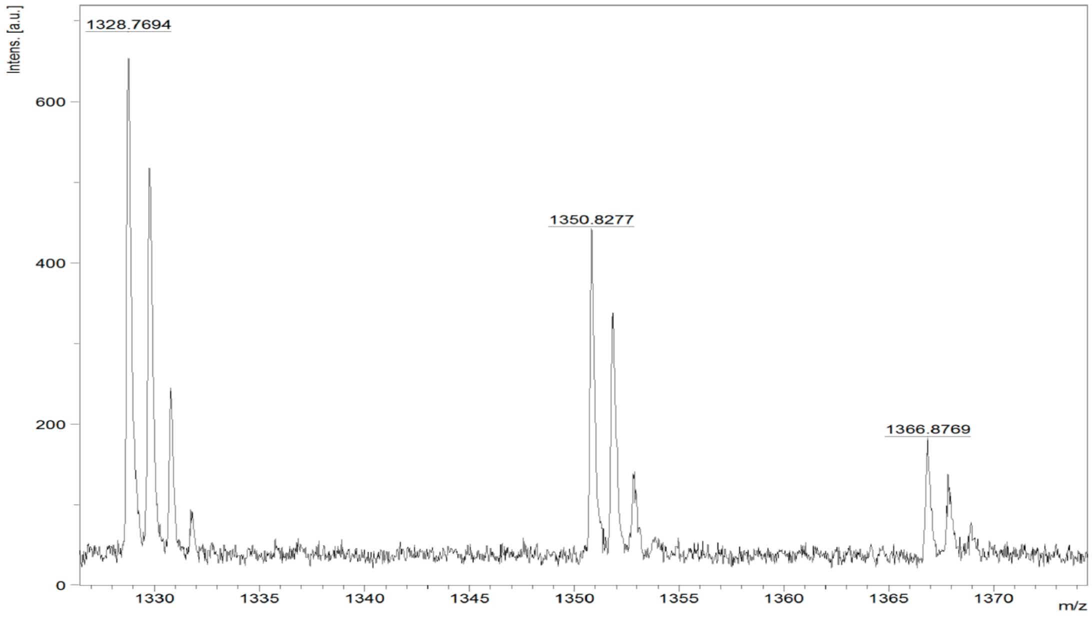

Supplement: Supplementary file 1 [file molecules-22-01632-s001.pdf]
